# Functional constipation and the effect of prebiotics on the gut microbiota: a review

**DOI:** 10.1017/S0007114522003853

**Published:** 2023-09-28

**Authors:** Rene Erhardt, Joanna E Harnett, Elizabeth Steels, Kathryn J Steadman

**Affiliations:** 1 School of Pharmacy, The University of Queensland, Brisbane, QLD 4102, Australia; 2 School of Pharmacy, The University of Sydney, Camperdown, NSW 2006, Australia; 3 Evidence Sciences, 3/884 Brunswick St, New Farm, QLD 4005, Australia

**Keywords:** Functional constipation, Prebiotics, Microbiota, Dietary fibre

## Abstract

Functional constipation is a significant health issue impacting the lives of an estimated 14 % of the global population. Non-pharmaceutical treatment advice for cases with no underlying medical conditions focuses on exercise, hydration and an increase in dietary fibre intake. An alteration in the composition of the gut microbiota is thought to play a role in constipation. Prebiotics are non-digestible food ingredients that selectively stimulate the growth of a limited number of bacteria in the colon with a benefit for host health. Various types of dietary fibre, though not all, can act as a prebiotic. Short-chain fatty acids produced by these microbes play a critical role as signalling molecules in a range of metabolic and physiological processes including laxation, although details are unclear. Prebiotics have a history of safe use in the food industry spanning several decades and are increasingly used as supplements to alleviate constipation. Most scientific research on the effects of prebiotics and gut microbiota has focussed on inflammatory bowel disease rather than functional constipation. Very few clinical studies evaluated the efficacy of prebiotics in the management of constipation and their effect on the microbiota, with highly variable designs and conflicting results. Despite this, broad health claims are made by manufacturers of prebiotic supplements. This narrative review provides an overview of the literature on the interaction of prebiotics with the gut microbiota and their potential clinical role in the alleviation of functional constipation.

## Graphical abstract

**Figure:**
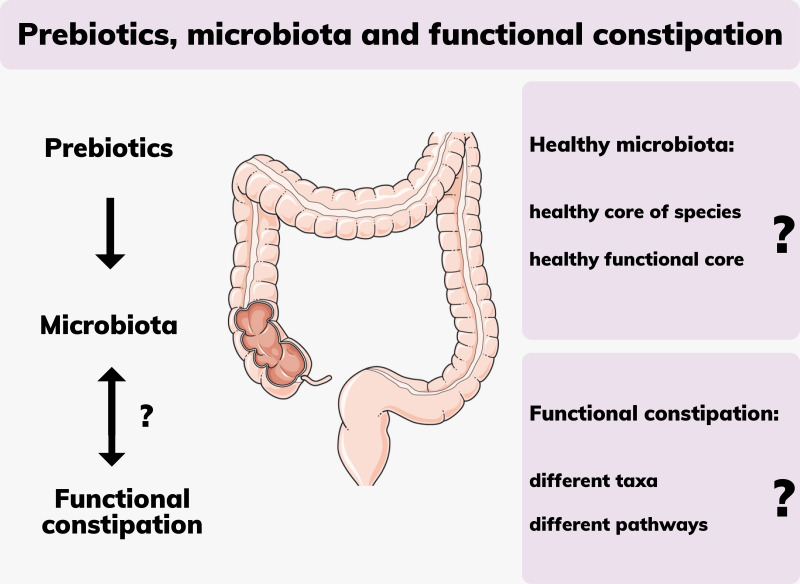

Functional constipation is the most common gastrointestinal disorder affecting about 14 % of the global population and it negatively impacts the quality of life of those affected^(1)^. In addition, there is a substantial cost to health care systems and further out of pocket costs are incurred by those suffering from the condition. In the UK, costs of £168 million to the National Health Service have been reported by the Bowel Interest Group for just 1 year (2018–2019), with more than 175 000 patient days spent in hospital and numbers are increasing^(2)^. Functional constipation is defined as infrequent bowel motions fewer than three times per week over a 3-month period with feelings of incomplete evacuation and excessive straining, without an underlying disease^(3)^. Non-pharmacological treatment advice from health care authorities such as the British Nutrition Foundation includes increasing fluid intake, physical activity and increasing dietary fibre consumption to a recommended daily intake of 30 g for adults^(4)^.

The importance of the gut microbiota for digestive health has been increasingly recognised in recent years and considerable research efforts and funding have been invested to elucidate the details^(5)^. An association has been made between the composition and function of the gastrointestinal microbiota in inflammatory bowel disease, coeliac disease, cancer, major depressive disorder and a range of extraintestinal disorders^(6,7)^. Less is known about an association between the gut microbiota and functional constipation^(8)^. Diet greatly influences the composition of these microbial communities and particular research attention has focussed on the effects of fermentable fibres that humans are unable to digest^(8)^. Various species of bacteria have been identified as utilising this fibre as fuel to produce metabolites that contribute to the energy balance of the host and confer colonic and extraintestinal health benefits such as production of B vitamins^(9)^. These fibres known as ‘prebiotics’ have also been in use for several decades as ingredients in manufactured food products as well as in nutritional supplements. However, the mechanisms of how they exert an effect on the microbial environment and the host have not been fully elucidated^(9)^.

This review seeks to summarise the literature pertinent to the topic of functional constipation and the microbiota and outlines key research gaps that require focussed scientific inquiry, namely, the intersect between prebiotics, the gut microbiota and the effect on constipation.

## Constipation

Functional constipation (Table 1) is a type of functional gastrointestinal disorder that is characterised by a combination of motility disturbance, visceral hypersensitivity, altered gut microbiota and altered central nervous system processing^(10)^. The causes for constipation are multifactorial and still under investigation. They are broadly characterised as primary idiopathic with normal transit, slow transit or evacuation disorder and secondary constipation which includes medication side-effects, obstruction, metabolic, neurological, psychiatric or systemic causes^(10)^. In clinical practice, the side effects of some medications can result in alterations to bowel movements including constipation. Medications associated with altered bowel movements include non-steroidal anti-inflammatory drugs, calcium channel blockers, opiates, anti-depressants, anticholinergic agents, diuretics, antacids and chemotherapy agents^(11)^.

**Table 1.** Terms and definitions of functional constipation, prebiotics and dietary fibre

Terms and definitions	
Functional constipation	Functional constipation, normal and slow transit constipation, chronic or chronic idiopathic constipation are used interchangeably^(14,69)^. Here the definition according to the ROME foundation’s expert committee of gastroenterologists is used, with the Rome IV criteria being current at the time of writing^(3)^. A person must have experienced at least two of the following symptoms for the previous 3 months to be diagnosed with functional constipation: Fewer than three spontaneous bowel movements per week; straining for more than 25 % of defecation attempts; lumpy or hard stools for at least 25 % of defecation attempts; sensation of anorectal obstruction or blockage for at least 25 % of defecation attempts; sensation of incomplete defecation for at least 25 % of defecation attempts; manual manoeuvring required to defecate for at least 25 % of defecation attempts. In addition, loose stools should rarely be present without laxatives and there should be no diagnosis of irritable bowel syndrome (IBS).
Prebiotics	In 2016, the International Scientific Association for Probiotics and Prebiotics defined prebiotics as substances which are selectively used by microbes conferring a demonstrated health benefit to the host^(70)^. They are mainly non-digestible oligosaccharides fructans, galactans and inulin^(71)^. They may include polyphenols and conjugated fatty acids. Candidate prebiotics include pectins, arabinoxylan, resistant starches, whole grains, cellulose, mannose, maltose, polydextrose, lactulose and β-glucans^(71)^.
Dietary fibre	Natural foods contain a mix of soluble and insoluble fibres. Soluble fibres can be viscous (psyllium) or non-viscous (guar gum), and in addition fermentable (guar gum, pectins, inulin) or non-fermentable (cellulose, lignin) with overlapping categories^(72)^. Only fermentable fibre has an effect on microbiota but not necessarily an effect on laxation^(70)^.

The negative impact on a person’s quality of life of this ailment is comparable to other chronic conditions such as dermatitis, chronic allergies, depression, diabetes or musculoskeletal conditions such as arthritis or osteoporosis, and the mental health effects are considered to be more severe than the physical components^(12)^. Adults and children have reported substantial limitations to their daily activities and only feel comfortable at rest. These negative impacts on quality of life extend to caregivers of children with constipation^(12)^.

**Fig. 1.**
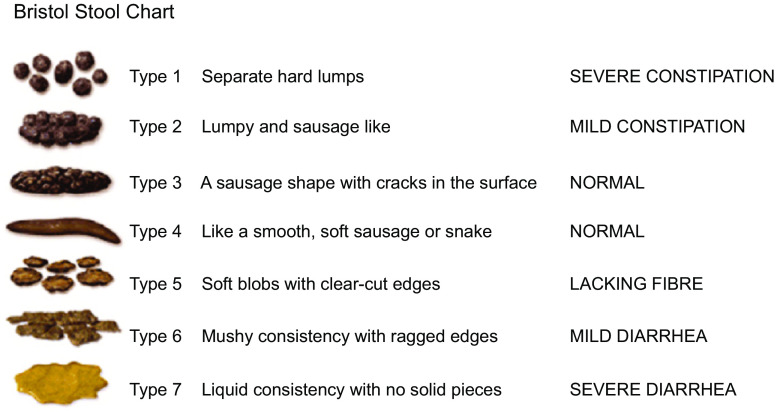
Bristol Stool Chart (Cabot Health, http://cdn.intechopen.com/pdfs-wm/46082.pdf). Commonly used classification tool for stools according to consistency.

For the patient, understanding the characteristics of a normal stool plays an important role in patient education and for monitoring and managing the treatment of functional constipation. The Bristol Stool Form Chart (Fig. 1) has been developed to describe stool consistency in an identifiable way and it has been validated to reliably indicate intestinal transit time^(13)^. It is broadly used in clinical practice and research for stool classification not only for irritable bowel syndrome (IBS) but also for functional constipation^(14–16)^. Type 1 is classified as severe constipation, type 2 as mild constipation, types 3 and 4 are normal, types 5 to 7 may indicate diarrhoea or urgency. This visual scale can be a valuable tool for integration into self-care and monitoring.

Standard management recommendations for constipation by health care authorities include exercising, adequate hydration and dietary changes with an increase of fibre intake^(17)^. Despite general agreement about these treatment recommendations, high quality clinical studies evaluating the efficacy of increased fibre intake in the management of functional constipation are lacking^(18)^. Due to the heterogeneity of definitions used for functional constipation, the use of the term dietary fibre without differentiation between non-fermentable fibre and prebiotics (Table 1), and the different types of cohorts studied, outcomes cannot easily be directly compared or generalised. Several meta-analyses of randomised controlled trials and Rome Foundation reports evaluating fermentable or non-fermentable fibre reported some benefits in the treatment of functional constipation, and they reported side effects such as increased flatulence^(19,20)^. Criticism of available studies includes concerns about selection bias, functional constipation being poorly defined or with large differences between studies and large placebo responses with differing endpoints^(21)^.

## Microbial composition and constipation

The causes of functional constipation are multifactorial, with dysbiosis of the gut microbiota considered to be one contributing factor^(20)^. Other factors may include low fluid intake, lack of dietary fibre, excess caffeine or alcohol intake, endocrine, neurological or psychological issues^(22)^. Differences in faecal microbial species and abundances have been reported for people with constipation compared to those with normal gut function, but data are limited and findings are very inconsistent between studies^(20)^.

For instance, two single point-in-time studies comparing the microbiota of healthy and constipated participants reported quite different results. Investigating the composition of the faecal microbiota, Mancabelli *et al.* ^(23)^ conducted a profiling analysis targeting the V3 region of the 16S rRNA gene in sixty-eight participants affected by constipation compared to seventy-nine healthy participants. Low counts of faecal *Bacteroides, Roseburia* and *Coprococcus* were found but species diversity in general was greater in constipated participants. Considering that an age-specific development and shift of the gut microbiota with substantial differences between children, adults and elderly have been observed, and the ages in the study ranged from 4 to 93 years, it has been suggested that subgroup analyses may have provided more specific information^(24)^. Shotgun sequencing was used to elucidate metabolic pathways in a subset of five constipated and five healthy participants. A high abundance of genes involved in methane production was identified in the constipated, whilst in healthy participants genes involved in carbohydrate and fatty acid metabolism were identified in greater abundance. The authors concluded their findings indicate an association between functional constipation and alterations of key microbial metabolic pathways, although they alert that the results from this small sample size require validation by larger studies.

In contrast, Parthasarathy *et al.* ^(25)^ compared faecal and mucosal microbiota samples of twenty-five healthy and twenty-five constipated females investigating different regions of the 16S rRNA gene (V3–V5 regions), finding characteristic differences between the microbial composition of the two specimen types. Unlike in Mancabelli *et al.* (2017), methane production was not associated with constipation. In faecal samples, the authors found the same taxa in both groups of participants, with differences only in abundance but not in species richness. It also has to be considered that this group of twenty-five constipated females consisted of thirteen with functional constipation, six with IBS-Constipation and six with moderate to very severe symptoms of mixed IBS, which can be expected to influence outcomes.

## Prebiotics

### Definition of a prebiotic

The definition of what constitutes a prebiotic had evolved since its inception in 1995 when it was restricted to non-digestible food ingredients that selectively stimulate the growth of a selected number of bacteria in the colon thought to be a benefit for host health, which limited it to fructo-oligosaccharides^(26)^. Other carbohydrates, such as resistant starches, pectin, gums and gluco-oligosaccharides were certainly recognised to be food for microorganisms, but they lacked specificity and they were excluded because of their potential to promote the growth of pathogens.

In the revised definition, a prebiotic selectively stimulates the growth of bacteria that have a ‘favourable’ metabolomic profile in the gastrointestinal tract^(27)^. Consequently, a wide range of plant components are considered to be prebiotics, such as inulin-type fructans, fructo-oligosaccharides and lactulose, which have been shown to increase colonies of microbial genera that are recognised to be health promoting, e.g. Lactobacilli and Bifidobacteria^(27)^. Prebiotics occur naturally in many fruits, vegetables and algae – it has been estimated that some 36 000 plant species contain fructo-oligosaccharides and other polysaccharides^(28)^. They are found in common foods such as asparagus, garlic, onion, wheat, honey, banana, barley, tomato, milk, peas and beans^(28)^. While prebiotics encountered in natural foodstuffs can be classified under dietary fibre, not all dietary fibre is prebiotic; fibre that is not fermentable by microbes is not considered to be prebiotic. Knowledge about prebiotic action in increasing colonic populations of beneficial commensal bacteria has been extrapolated to the assumption that combining probiotics with prebiotics into a synbiotic would confer an increased benefit^(29)^. A range of synbiotics is commercially available, for example *Lactobacillus* spp. and inulin, or *Lactobacillus, Streptococcus* and *Bifidobacterium* spp. and fructo-oligosaccharides.

The narrow definition of a prebiotic continues to be debated, with critics arguing that it is still unclear which microbes are relevant to human diseases, and although dysbiosis accompanies many diseases, the questions about causal relationships are largely unanswered^(30)^. Therefore, microbial selectivity as a criterion becomes questionable since there is no consensus about what constitutes a healthy microbiota. Molecular studies have shown that no single carbohydrate is likely to be fermented by only a selective group of microbes and none is fermented by all^(30)^. Cross-feeding, where metabolites from one bacterial strain create a niche for another, also contributes to greater diversity which is considered an indicator of good health^(31)^. Diet, lifestyle and genotype of the host have been shown to create an environment where microbes normally considered to be beneficial can become detrimental, bringing into question the over-simplistic notion of ‘good *v.* bad’. Thus, knowledge of functional effects of the gastrointestinal microbiota and prebiotics is needed to support the concept of a ‘prebiotic effect’^(30)^.

### The gut microbiota and prebiotics

The gut microbiota, with an estimated 3 million genes, is 150 times larger than the human genome and contributes to such an extent to our metabolic capacity that it is considered by many as fulfilling the functions of another organ^(32,33)^. The microbes produce enzymes that can ferment prebiotics, which humans otherwise have no capacity to metabolise. The effect of this fermentation is of interest since it provides a major energy source for the host and it produces metabolites that interact with the enteric nervous system, influencing motility^(34)^. In regards to constipation, prebiotics are a common plant-based food supplement employed to restore homeostasis of a dysbiotic gut^(8)^. Prebiotic fermentation and resulting metabolites, including short-chain fatty acids (SCFA), have far-reaching physiological consequences. SCFA play an important role as signalling molecules and their regulatory function for local, intermediary and peripheral metabolism has gained research interest since the discovery of receptors across a wide range of cells and tissue types around the body^(35)^. In the distal and innervated colon, SCFA appear to have a regulatory function on motility and defecation reflexes, and different prebiotics have different effects on microbial abundances^(34,35)^.

In addition, the individual microbial composition of each person is of great importance since bacterial species vary in their capacity to produce SCFA from different types of fibre. The optimal amount of SCFA has not been established, and most are absorbed and used by enterocytes or enter the circulation, so amounts measured in faeces may not necessarily reflect SCFA production rates^(36)^. SCFA play a role in the immune response via regulating differentiation and activation of immune cells and through regulation of inflammatory cytokines they act as key mediators of inflammation^(35)^. SCFA also modulate T-cell differentiation and inhibit inflammatory IFN-*γ*-producing cells. This role is considered by some as the key molecular link between diet, microbiota and health^(35)^. As discussed, the fermentation of prebiotics is a complex process and depending on the individual microbial composition of each person and the type of fibre, SCFA production rates may vary considerably between individuals^(37)^.

### Microbiota–gut–brain axis

**Fig. 2.**
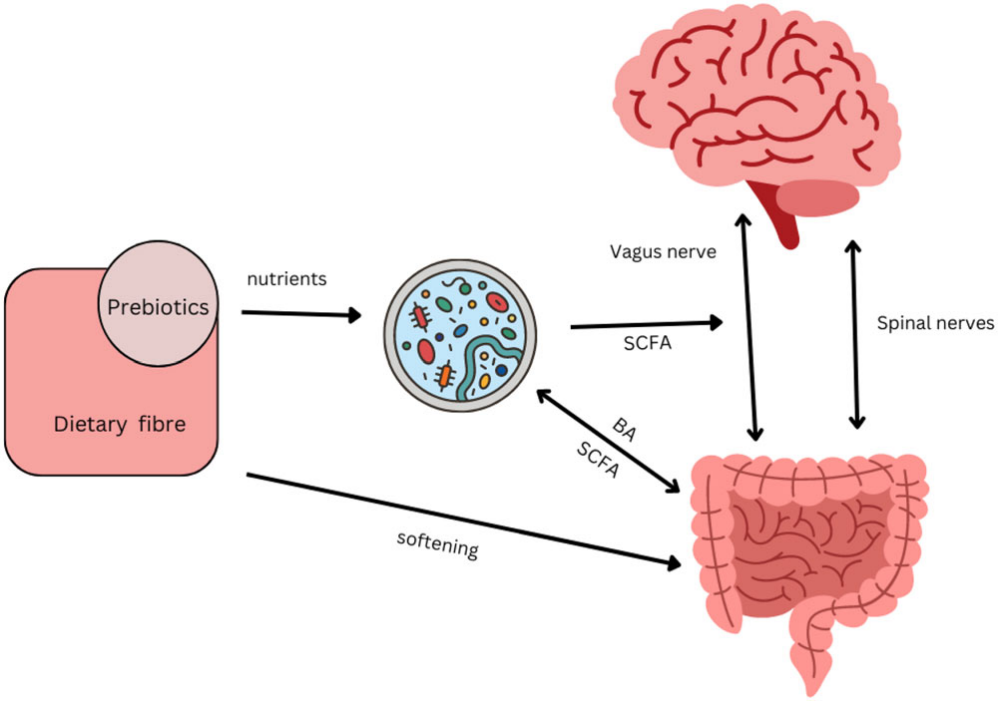
Interacting pathways between microbiota, prebiotics, dietary fibre, gut and brain affecting constipation. Several pathway impact on gut motility. There is bidirectional communication between gut and brain via vagal and spinal nerves, with serotonin as the main neurotransmitter^(34)^. SCFA produced by the microbiota can directly stimulate nerves cells in the gut, or indirectly stimulate enteroendocrine cells to produce serotonin and consequently trigger peristaltic reflexes^(34)^. SCFA can also directly stimulate receptors on the vagus nerve^(41)^. Secondary bile acids (BA) produced by the microbiota can affect motility, while BA themselves affect the composition of the microbiota^(42)^. The microbial composition of the gut is influenced by the enteric nervous system through immunological defence secretions, permeability or motility^(41)^. Transit time influences the composition of the microbiota through exposure to water and nutrients. Prebiotics such as galacto-oligosaccharides can increase SCFA-producing *Bifidobacterium* spp. and increase motility in constipated individuals^(51)^. Non-fermentable dietary fibre such as coarse wheat bran and psyllium husks soften stools making them easier to pass by mechanisms that do not directly involve the microbiota^(68)^ but affects the environment in which they reside.

Historically, functional gastrointestinal disorders have been investigated in terms of interactions between the enteric neuromuscular system, neurotransmitters and the brain, which gave rise to the term ‘gut-brain axis’^(38)^. More recent research identified a regulatory function of the brain on functional intestinal cells via neural and hormonal pathways^(39)^. At the same time, the influence of commensal bacteria as well as pathogens on the brain has been established, leading to the extension of the model now named the ‘microbiota–gut–brain axis’^(40)^. The brain and microbiota are thought to communicate via the immune system, the vagus nerve, tryptophan metabolism and the enteric nervous system, through microbial metabolites such as SCFA, branched-chain amino acids and peptidoglycans. Metabolites of the gut microbiota, in particular the neurotransmitter serotonin produced by *Clostridium* spp. and *Escherichia coli,* have been shown to exert a modulatory effect on peristalsis and motility through interactions with the enteric nervous system^(41)^. In addition, ‘cross-talk’ between bile acids and the microbiota regulates motility: bile acids themselves affect the composition of the microbiota whilst deconjugated bile acids are used as signalling molecules by the microbiota^(42)^ (Fig. 2). Research showed that serotonin can also influence microbial composition of the gut: relative abundances of receptive species, such as *Turicibacter* are increased^(43)^. This, in turn modulates functional capabilities with increases in steroid and lipid metabolism, but details require further investigation.

### Studies evaluating clinical and microbial effects of prebiotics in people with constipation

A limited number of clinical trials have investigated the efficacy of prebiotics for alleviating constipation and the concomitant effects on the microbiota. The comparison of studies and the evaluation of prebiotic efficacy is seriously hampered by a number of issues plaguing the field. Only some studies have used people with symptoms of constipation^(44–51)^ (Table 2), with differing criteria of functional constipation ranging from undefined or vague^(23,44,46,47,50)^, Rome I, III or IV^(25,45,51)^, or combinations with IBS-C criteria^(49)^, while others tested healthy people^(52–59)^. The number of participants varies greatly and very small studies lack the power for generalisable results^(60,61)^. There are large differences in age groups of study participants between studies, with some sampling from a very wide range^(23,44)^. The products/formulations and dosages vary considerably between studies, from 1 g/d^(52)^ to 20 g/d^(45)^ in studies on inulin, or single ingredient studies^(46)^ through to formulations that include multiple ingredients such as inulin, lactitol and aloe vera^(48)^, or without listing ingredients at all^(50)^. Finally, every study differs in the methodology used for microbial analysis^(23,44–47,49,51)^ and often a restricted range of microbes were investigated, for example, five particular species^(47)^ or only *Lactobacillus* spp.^(44)^ or *Bifidobacterium* spp.^(46)^. This leads to an equivocal overall picture of the efficacy of prebiotics, which is particularly lamentable since functional constipation is of such a high prevalence in the general population. Nonetheless, the trials that have been reported do provide a wealth of information.

**Table 2.** Summary of clinical trials in participants with functional constipation that evaluated the effect of prebiotic treatment on bowel frequency and composition of the gut microbiota

Authors	Title	Product, dosage, duration, participants	Study type	Outcome
Takahashi *et al.* 1994^(44)^	Influence of partially hydrolysed guar gum on constipation in women	PHGG 11 g/d 9 weeks Fifteen women (18–48 years) with constipation	Open label, 3 weeks no treatment, 3 weeks PHGG, 3 weeks no treatment	Increase in stool frequency to 4·4 motions/week; doubling of *Lactobacillus* spp. during intervention
Bouhnik *et al.* 2004^(45)^	Prospective, randomised, parallel-group trial to evaluate the effects of lactulose and polyethylene glycol-4000 on colonic flora in chronic idiopathic constipation	Lactulose or polyethylene glycol (PEG) 20 g/d 4 weeks Sixty-five adults (mean age 58 years) with constipation	Open label, randomised, parallel groups, *n* 33 lactulose, *n* 32 PEG; 20 g/d for 1 week, then individual variation according to efficacy and tolerance 10–30 g/d	Increase in bowel motions to 7·8 motions/week in lactulose and 6·8/week in PEG group; increase in *Bifidobacterium* spp. in lactulose group, decrease in SCFA production in PEG group
Marteau *et al.* 2011^(46)^	Effects of chicory inulin in constipated elderly people: a double-blind controlled trial	Inulin 15 g/d 4 weeks Fifty adults (51–62 years) with constipation	Double-blind, randomised, parallel groups, *n* 25 inulin, *n* 25 maltodextrin	Reduced defecation difficulties; increase in *Bifidobacterium* spp. and total bacterial count with inulin
Linetzky Waitzberg *et al.* 2012^(47)^	Microbiota benefits after inulin and partially hydrolised guar gum supplementation – a randomised clinical trial in constipated women	Inulin/PHGG blend 15 g/d 3 weeks Thirty-two women (36–40 years) with constipation	Double-blind, randomised, parallel groups, *n* 14 inulin/PHGG blend, *n* 18 maltodextrin	Increase in stool frequency to 3·9 motions/week with inulin/PHGG and 3·2 with maltodextrin; *Clostridium* spp. count reduced with inulin/PHGG blend; no difference in SCFA production
Chu *et al.* 2019^(48)^	Prebiotic UG1601 mitigates constipation-related events in association with gut microbiota: A randomised placebo-controlled intervention study	inulin, lactitol, aloe vera mix 13 g/d 4 weeks forty adults with constipation (21–51 years)	Double-blind, randomised, parallel groups, *n* 20 prebiotic, *n* 20 maltodextrin	Increase of bowel motions in both groups to 4·1/week; *Roseburia* increased and *Lachnospiraceae* decreased in prebiotic group; no difference in SCFA concentration between groups
Jalanka *et al.* 2019^(49)^	The effect of psyllium husk on intestinal microbiota in constipated patients and healthy controls	Psyllium 21 g/d 18 d, Sixteen adults with constipation (mean age 41 years ± 15·7 years) were compared to a separate trial involving nine healthy adults (26 years ± 4·1 years)	Double-blind, randomised crossover, 6 d high dose, 6 d maltodextrin with 1 week washout between.	Improvement in bowel function; significant changes in microbiota; increased *Lachnospira, Faecalibacterium, Phascolarctobacterium, Veillonella, Sutterella and decreased Coriobacteria and Christensenella*
Stachowska *et al.* 2022^(50)^	Improvement of bowel movements among people with a sedentary lifestyle after prebiotic snack supply - preliminary study	Unspecified prebiotic soluble fibre in snack bar high dose 13·9 g/d or low dose 10 g/d 14 d, twenty adults with constipation (mean age 40·6 years ± 5·4 years)	Double-blind, randomised, parallel groups, *n* 10 high dose, *n* 10 low dose	Increase of bowel motions in both groups to 6/weeks; increase in SCFA production in both groups
Schoemaker *et al.* 2022^(51)^	Prebiotic galacto-oligosaccharides impact stool frequency and fecal microbiota in self-reported constipated adults: A randomised clinical trial	Galacto-oligosaccharide low dose 5·5 g/d or high dose 11 g/d; 3 weeks 132 adults with constipation (25–52 years)	Double-blind, randomised, parallel groups, *n* 45 low dose, *n* 44 high dose, *n* 43 maltodextrin	Significantly higher increase in bowel motions with high dose for sub-group with low stool frequency at baseline; Higher *Bifidobacterium* and *Anaerostipes hadrus* in high dose group

PHGG, partially hydrolysed guar gum.

*Bifidobacterium* is a genus that is commonly associated with a healthy gut and is often highlighted by studies investigating an association between gut function and microbiota. In separate trials, faecal Bifidobacteria increased with lactulose^(45)^ and inulin^(46)^ consumption in participants afflicted by functional constipation, and this was accompanied by improved bowel function. Increases in Bifidobacteria and stool frequency were also reported in trials with healthy participants who took lactulose^(52)^, while for inulin Bifidobacteria increased but these healthy participants did not experience changes in stool frequency^(58)^. Bifidobacteria also increased in healthy people, again without a change in stool frequency, with the use of arabinogalactans^(56,57)^ or galacto-oligosaccharides^(54,55)^. Schoemaker *et al.* (2022) also found that galacto-oligosaccharides increased *Bifidobacterium* spp. but highlighted the importance of working with participants actually suffering with constipation, as they found a significant increase in stool frequency only on sub-group analysis of participants with a particularly low frequency of bowel motions at the start of the trial^(51)^.

In contrast, no difference in *Bifidobacterium* spp. was found between two groups of thirty females with constipation who either used a blend of inulin and partially hydrolysed guar gum or maltodextrin for 21 d^(47)^. Both groups had increases in bowel motions, with no significant difference between groups. A decrease in pathogenic *Clostridium* spp. in the prebiotic group and an increase thereof in the maltodextrin group was observed^(47)^. Similarly, no changes in Bifidobacteria were found in participants taking psyllium husk, whether healthy or constipated, and both groups experienced improved bowel function^(49)^. The authors analysed the V4–V5 regions of the 16S rRNA gene and found that there was a significant effect of the prebiotic on the composition of the microbiota in both groups of participants, although more pronounced in the constipated participants. Here, a significant increase in SCFA producers *Lachnospira, Faecalibacterium* and *Roseburia* was identified. These studies show that although Bifidobacteria are generally considered to be beneficial, an increase thereof is not necessarily correlated with an increase in stool frequency.

In health research, increasingly sophisticated bioinformatic technology is employed to predict bacterial genera associated with various illnesses including constipation^(62)^. Machine learning technology is a discipline of computer science where computers are programmed to be able to recognise patterns from data following mathematical rules and statistical assumptions to enable the development of predictive models from a dataset. In a meta-analysis of five research cohorts with 3056 faecal amplicon sequence data, Chen *et al.* ^(62)^ employed systematic machine learning technology to identify potential biomarkers for constipation. The model they constructed enabled the identification of fifteen key genera as possible biomarkers with the most significant being *Serratia, Dorea, Agathobacter, Hungatella* and *Aeromonas*. In addition, the taxonomic analyses by Chen *et al.* ^(62)^ also found greater species diversity and richness in the constipated group. Interestingly, the commonly investigated genera *Bifidobacterium* and *Lactobacillus* did not feature on their list.

The Rome Foundation stated that quantitative and qualitative changes in mucosal and faecal microbiota have been encountered in functionally constipated patients, with greater changes in IBS compared to those affected by functional constipation^(10)^. The researchers recognised equivocal results reported from several studies and recommended larger clinical trials with clearer target definitions and a closer cooperation between experienced clinical researchers and microbial ecologists, highlighting the importance of interdisciplinary research contributions to trial design and interpretation.

In summary, researchers agree that prebiotic products affect the microbiota but not all act in the same way in every person. There is some data on clinical utility for the use of prebiotics in constipation, but it is inconclusive. Apart from the necessity for large-scale clinical trials, the mechanisms of action of prebiotics need further elucidation. The microbial composition typically encountered in individuals with functional constipation also requires further investigation; although there is some evidence that alterations to the composition of the gut microbiota may contribute to the symptoms^(10,18,23)^, constipation creates a habitat that favours microbes able to proliferate in an environment with longer exposures to water and nutrients^(63)^ and large cohort studies have demonstrated that diet and transit time influence composition of the gut microbiota^(64,65)^. Consequently, determining whether changes in the microbiota have any role in cause or are simply an effect of constipation is exceptionally complex to determine.

### Challenges with assessing the gastrointestinal microbiota composition

Despite considerable advances in research, there is still no well-established definition of what constitutes a normal healthy gastrointestinal microbiota^(66)^. Concerted research efforts, including the two major collaborative studies, MetaHIT and Human Microbiome Project, have identified the most dominant bacterial communities of the human gut microbiota to genus or species levels^(5,67)^. This does provide us with an inventory list of ‘who is there’, but what the functions of these bacterial genes encode for remains largely unknown. While a core gastrointestinal microbiota is thought to exist, large site-specific intra- and inter-individual variations have been observed^(66)^. Critics of the concept of a common ‘core of species’ constituting a healthy microbiota would like to see it replaced with that of a healthy ‘functional core’^(66)^. The healthy gut microbiota needs to be able to maintain stability, resist major change induced by diet, pathogens or drugs, and be able to recuperate after having been impacted upon by stressors or perturbations^(66)^. Since a stable, balanced microbiota is considered the cornerstone for a healthy gut, a maladaptive state of imbalance characterises an unhealthy one. This has been described as a state of dysbiosis, which can be broadly described as any change to the composition of resident commensal communities relative to the community found in healthy individuals. Functional constipation may be viewed as a consequence of dysbiosis^(66)^.

## Summary

Prebiotics are widely used in clinical practice and more so in the food industry but overall, there is a paucity of evidence from high-quality clinical trials regarding the quality, efficacy and safety of prebiotic formulations used in the management of constipation. Heterogenous study designs and variations in prebiotic types have contributed to a lack of generalisability and impeded the potential for direct application in clinical practice in terms of a diagnostic or therapeutic use. Despite these limitations, the overall body of research indicates a potential role for a range of prebiotic types in the management of constipation. Larger prospective studies with longer intervention periods and multiple timepoints are needed to elucidate the association between changes in the microbiota with gut motility in individuals with constipation. Multiple timepoints of sampling would contribute to understanding the extent of the effects of diet on the composition of the microbiota. In addition, cross-over studies could provide valuable insights into individual responses to changes in dietary fibre intake. Clinical trials designed as complete feeding studies with single ingredient modifications would greatly improve our understanding of the potential benefits of prebiotics and contribute to understanding whether an alleviation of constipation is associated with changes in the microbiota towards that of healthy controls.
